# Manometric Measurement of the Sphincter of Oddi in Patients with Common Bile Duct Stones: A Consecutive Study of the Han Population of China

**DOI:** 10.1155/2017/9031438

**Published:** 2017-01-04

**Authors:** Yadong Feng, Jie Zhang, Chunhua Jiao, Hong Zhu, Wenfang Cheng, Shunfu Xu, Bin Xiao, Jinliang Ni, Xiaoxing Chen

**Affiliations:** ^1^Department of Gastroenterology, First Affiliated Hospital of Nanjing Medical University, 300 Guangzhou Road, Nanjing 210029, China; ^2^Department of Gastroenterology, Zhongda Hospital, School of Medicine, Southeast University, 87 Dingjiaqiao, Nanjing 210029, China; ^3^Department of Radiology, Xuzhou Central Hospital, 99 South Jiefang Road, Xuzhou 221006, China; ^4^Pancreatic Centre, First Affiliated Hospital of Nanjing Medical University, 300 Guangzhou Road, Nanjing 210029, China

## Abstract

*Objective*. Role of dysfunction of the sphincter of Oddi (SO) in choledocholithiasis is controversial. This study was to evaluate SO motor activity in patients with common bile duct (CBD) stones in the Han population of China.* Patients and Methods*. In this study, 76 patients with CBD stones were enrolled in a single tertiary endoscopy center. Data of SO motor activities was prospectively evaluated by endoscopic manometry. Mean basal SO pressure, amplitude, and frequency were collected and analyzed.* Results*. The mean basal SO pressure, amplitude, and frequency were 52.7 ± 40.0 (1.60–171.1) mmHg, 39.9 ± 19.7 (14.9–115.5) mmHg, and 5.7 ± 3.2 (1.3–13.8)/min, respectively. The basal SO pressure was higher in patients with CBD stones < 10 mm in diameter than that in those with CBD stones larger than 10 mm in diameter (60.7 ± 41.0 mmHg versus 36.8 ± 29.4 mmHg, *P* = 0.043). There was no significant difference in the basal SO pressure, amplitude, and frequency when compared with the CBD diameter, CBD stone number, prior cholecystectomy, periampullary diverticula, and symptoms. Levels of alanine aminotransferase, aspartate transaminase, γ-glutamyl transpeptidase, and alkaline phosphatase showed no significant difference in patients with normal or elevated basal SO pressure.* Conclusion*. These results identify that, in Chinese Han population, abnormalities of SO motor activity are associated with CBD stones.

## 1. Introduction

The presence of common bile duct (CBD) stones is a prevalent worldwide disorder. Secondary CBD stones are more common in Western countries, while primary stones occur more frequently in Asia [1, 2]. It has been reported that primary CBD stones are mostly pigment stones, while secondary CBD stones are predominantly cholesterol stones [3]. The pathogenesis of primary CBD stones may differ from that of secondary CBD stones.

Although the pathogenesis of CBD stones is not fully known, some risk factors for choledocholithiasis [3], such as increasing age, chronic bile duct inflammation, impaired intestinal barrier, and duodenal diverticulum, and some biological and behavioral factors are associated with bile duct stone formation. Biliary stasis is an important parameter for CBD stone formation, especially in primary CBD stones [3, 4]. The basal pressure of the sphincter of Oddi (SO) is higher than that at the end of the bile duct and the intracavity pressure of the duodenum. It has been suggested that SO dysfunction may cause a disorder of the biliary stasis [5] and may be involved in patients with CBD stones in Western countries [6–9]. According to the study by de Masi et al. [6], the motor activity of SO did not play a significant role in the formation and/or retention of CBD stones. However, more data suggested that abnormality in the SO motor function was related to the formation of CBD stones [7–9]. It should be further discussed because of this discrepancy. To our knowledge, comparable data on the relationship between SO motor activity and CBD stones from Asia are lacking. Because of an extremely high prevalence of primary duct stones in Eastern Asia [10], it is important to know if SO dysfunction is involved in patients with CBD stones.

The etiology of CBD stones in Asia, including China, remains to be further investigated. Whether disorder of SO was involved in Chinese patients with CBD stones, which are mostly primary brown stones, has not been reported. Therefore, we enrolled patients with CBD stones in Han population, which is about 95% among Chinese, and prospectively evaluated the motor activity of SO in one tertiary endoscopy center in China.

## 2. Materials and Methods

### 2.1. Ethics Approval

The study was approved by the Ethics Committee of the First Affiliated Hospital with Nanjing Medical University (Nanjing, China). The inclusion criteria were as follows: (1) an age of 18–80 years, (2) CBD stones confirmed by type B ultrasonic test, computed tomography, or magnetic resonance cholangiopancreatography, and (3) patients with the first endoscopic retrograde cholangiopancreatography (ERCP) session for CBD stone removal. The exclusion criteria were as follows: (1) acute cholangitis or septic shock, (2) a history of previous endoscopic sphincterotomy (EST) or endoscopic papillary balloon dilatation (EPBD), (3) coagulopathies (platelet count < 50,000 × 10^3^/*μ*L or an international normalized ratio > 1.5), (4) malignant biliary obstruction, (5) a Billroth II, Roux-en-Y, or choledochotomy anatomy, (6) failure of biliary cannulation or a requirement of precut sphincterotomy, or (7) refusal to undergo SO manometry. All enrolled patients signed a written informed consent before the ERCP.

This study was prospectively performed at the First Affiliated Hospital of Nanjing Medical University (Nanjing, China). From March 2013 to January 2016, a total of 76 patients were enrolled in the study (43 males and 34 females, 36 with prior cholecystectomy, 18 with previous pancreatitis, and ranging in age from 24 to 78 years). Data on liver function before ERCP were also recorded.

### 2.2. Protocol of SO Manometry

A duodenoscope (JF-260V, Olympus, Tokyo, Japan) and a high resolution manometry system (ManoScan360, Given SSI, USA) were used for SO manometry (SOM) in the ERCP procedure. Drugs that affected SO motility, such as calcium antagonists, anticholinergics, and parasympathomimetics were avoided for 48 h. All patients were under sedation by intravenous injection of propofol, which does not affect SO motility [11]. Cannulation of the bile duct was attempted using a sphincterotome (Olympus, Tokyo, Japan) with a 0.035-inch guide wire (Boston Scientific, Natick, MA, USA). A 0.018-inch guide wire (Wilson-Cook Medical, Winston-Salem, NC, USA) was introduced and the sphincterotome was withdrawn. A sleeve SOM catheter (Mui Scientific, Toronto, Canada) was introduced into the papilla along the fine guide wire. SOM was done according to the manometry instructions. Duodenal pressure was used as a zero reference. The SO basal pressure, amplitude, and frequency were recorded for 5 min.

### 2.3. Endoscopic Procedures for Extraction of CBD Stones

After the SOM, contrast medium was injected into the CBD for a cholangiogram. EST or EPBD following lithotomy was performed. Information on periampullary diverticulum (PAD), CBD diameter, CBD stone size, and CBD stone number was recorded in the same ERCP session. Post-ERCP complications were evaluated according to previously established criteria [12].

### 2.4. Statistical Analysis

All data are expressed as the mean (*X*) ± standard deviation (SD). The chi-square test, analysis of variance (one-way ANOVA), Student's* t-*test, and the Mann–Whitney *U* test were performed using SPSS statistical software for Windows, version 16.0 (SPSS, Chicago, IL, USA). A *P* value < 0.05 was considered to be statistically significant.

## 3. Results

Four cases of hyperamylasemia, three cases of mild pancreatitis and one moderate case of pancreatitis were present in the patient cohort. There was no hemorrhage, perforation, or cholangitis. The mean basal SO pressure, amplitude, and frequency of all patients were 52.68 ± 40.03 (1.60–171.1) mmHg, 39.93 ± 19.67 (14.9–115.5) mmHg, and 5.73 ± 3.20 (1.3–13.8)/min, respectively. The results of stratified analyses involving CBD diameter, CBD stone number and size, prior cholecystectomy, periampullary diverticulum, and symptoms are listed in Table 1. There was no significant difference in basal SO pressure, amplitude, and frequency compared with the CBD diameter, CBD stone number, prior cholecystectomy, PAD, and symptoms. The mean basal SO pressure in patients with CBD stones ≤ 10 mm was significantly higher than in those with larger CBD stones (60.69 ± 40.67 mmHg versus 36.75 ± 29.40 mmHg; *P* = 0.043). The differences of SO amplitudes and frequencies were not significant. The levels of alanine aminotransferase (ALT), aspartate transaminase (AST), *γ*-glutamyl transpeptidase (*γ*-GT), and alkaline phosphatase (AKP) were not significantly different in patients with normal or elevated basal SO pressure (Table 2).

## 4. Discussion

The formation and development of choledocholithiasis have not been completely characterized. An altered SO motor pattern might contribute to the development of choledocholithiasis [7, 15]. Few studies have been reported in the past 30 years, and the role of SO motor activity in CBD stones is still inconsistent [6–9, 13, 15, 16]. The objective of the present study was therefore to evaluate the pressure of SO in patients with CBD stones in the Chinese Han population, which is the most majority ethnic group in China.

In this study, we did not evaluate SO motor activity in healthy people for ethical reasons. In our center, patients without CBD stones are rarely subjected to ERCP. So we did not set a control group which maybe consisted of healthy people and/or patients without CBD stones. According to the study by Gregg and Carr-Locke [13], basal SO pressure of bile duct in healthy volunteers was 13.4 ± 6.2 mmHg. Available data on basal SO pressure, amplitude, and frequency in healthy volunteers [13, 14] were listed in Table 3. These were used as references, although those were from Western countries. Our results showed a significantly increased basal SO pressure value of 52.68 ± 40.03 mmHg in the enrolled patients. Yuan et al. [17] reported that the basal SO pressure values in two groups of Chinese patients with choledocholithiasis were 30.88 ± 16.11 and 27.80 ± 15.88 mmHg. In a study by Yang et al. [18], values of basal SO pressure, amplitude, and frequency were not reported. Our data showed mean basal SO pressure higher than 40 mmHg and indicated possible roles of SO dysfunction in patients with CBD stones. Previous studies [7, 8] reported increased basal SO pressure in patients with CBD stones, suggesting a correlation between SO dysfunction and CBD stones. In patients with choledocholithiasis, SO dysfunction induced increased pressure within the common bile duct and caused colicky pain [19]. In our study, most majority of patients experienced abdominal pain. However, basal SO pressure did not differ with different symptoms, and the levels of ALT, AST, *γ*-GT, and AKP, which represent biochemical characteristics of biliary obstruction, did not vary with different basal SO pressure. Increased basal SO pressure may inhibit bile flow into the duodenum and facilitate bile storage in the bile duct [5], thus promoting stone formation. The SO amplitude and frequency were normal, with no difference in all patients. Our results are consistent with two previous studies [5, 8], reporting no variation of SO amplitude and frequency in patients with choledocholithiasis. However, according to previous reports [6–8, 13, 15, 16], the possible relationship between SO motor activity and CBD stones remains controversial and needs further study.

A CBD stone not larger than 10 mm is easy for extraction, and we performed stratified analyses with CBD sizes at 10 mm in diameter. Based on our results, basal SO pressure in patients with CBD stones ≤ 10 mm in diameter was greater than that in patients with CBD stones > 10 mm. Biliary microlithiasis and crystals, which are not imaged by conventional ultrasonography or cholecystography, contribute to SO dysfunction [19]. Microlithiasis may exist in patients with CBD stones regardless of the functional status of the SO [20]. There was no difference of SO motor activity between CBD stone numbers, so we suggest that SO dysfunction is in the early stages of CBD stone formation. However, whether the increased basal SO pressure in CBD stone formation is the cause or the affect remains to be further explored.

It has been reported that SO motor activity was not influenced by variations in the diameter of the common bile duct or by previous cholecystectomy [6]. In our study, SO motor activity did not vary with the CBD diameter or by prior cholecystectomy.

PAD is a risk factor for CBD stone formation that often causes a dilated CBD [21] that may affect the function of the SO [22]. In our study, about 36.8% (28/76) patients were with PAD, and this is similar to the result of a recent study from South China that the percentage is 38.8% (99/255) [23]. Although PAD may be an important factor for the occurrence of CBD stones, there has been no previous report on the relationship between SO motor function and PAD. In our study, there was no difference of SO motor function between patients with or without PAD. Our results are similar to the study by Skalicky [24] that there is no correlation between PAD and symptoms in patients with CBD stones. PAD-induced pancreatobiliary reflux [25] may be the main reason for the increased incidence of CBD stones [26].

Previous studies have suggested that SOD causes choledochal cysts, thus contributing to the formation of bile duct stones [21, 27]. Our results showed the involvement of SO dysfunction in patients with CBD stones and demonstrated the feasibility of EST in ERCP. Yang et al. [18] reported that, instead of weakened SO function after papillotomy [15], biliary infection was a main risk factor for long-term complications after EST.

In conclusion, our findings suggest that SO dysfunction is associated with CBD stones in Chinese Han patients, who are mostly with primary brown stones. This study may therefore contribute to our knowledge of the etiology of CBD stones. In ERCP session for removal of CBD stones, EST or EPBD is essential, which induces decreased SO pressure. In future, we may evaluate the recurrence of CBD stones in those who received endoscopic lithotomy and with lower basal SO pressure.

## Figures and Tables

**Table 1:** Analysis of SO motor activity stratified by CBD diameter, CBD stone numbers and sizes, prior cholecystectomy, periampullary diverticulum, and symptoms.

Factors	SO motor activity		
	Basal SO pressure (mmHg)	Amplitude (mmHg)	Frequency (/min)
CBD diameter			
≤10 mm (*n* = 25)	56.34 ± 41.13	38.23 ± 19.67	5.22 ± 3.29
>10 mm (*n* = 51)	56.24 ± 39.89	40.77 ± 20.68	5.99 ± 3.15
*P* value	0.992	0.600	0.333
CBD stone numbers			
Single (*n* = 52)	60.31 ± 43.24	41.76 ± 18.79	6.31 ± 3.37
Multiple (*n* = 24)	47.53 ± 31.04	35.98 ± 21.30	5.49 ± 2.31
*P* value	0.198	0.236	0.120
CBD stone size			
≤10 mm (*n* = 62)	60.69 ± 40.97	39.49 ± 19.64	5.60 ± 3.28
>10 mm (*n* = 14)	36.75 ± 29.40	41.90 ± 20.37	6.35 ± 2.83
*P* value	0.043	0.683	0.443
cholecystectomy			
Yes (*n* = 36)	48.23 ± 36.31	42.02 ± 24.90	5.62 ± 2.81
No (*n* = 40)	63.52 ± 42.25	38.06 ± 13.40	5.84 ± 3.54
*P* value	0.097	0.383	0.775
PAD			
Yes (*n* = 28)	58.65 ± 44.62	40.03 ± 18.03	5.37 ± 3.01
No (*n* = 48)	54.89 ± 37.53	39.88 ± 20.74	5.95 ± 3.31
*P* value	0.696	0.975	0.452
Main symptoms			
Colicky pain (*n* = 58)	57.47 ± 42.91	40.55 ± 20.20	6.00 ± 3.46
Jaundice (*n* = 8)	40.42 ± 27.06	31.35 ± 14.48	4.47 ± 1.60
Symptoms-free (*n* = 10)	69.07 ± 29.20	41.01 ± 21.02	5.24 ± 2.29
*P* value	0.475	0.683	0.396

SO: sphincter of Oddi, CBD: common bile duct, PAD: periampullary diverticula.

No significant difference in basal SO pressure, amplitude, and frequency compared with the CBD diameter, CBD stone numbers, prior cholecystectomy, periampullary diverticula (PAD), and symptoms. The mean basal SO pressure in patients with CBD stones ≤ 10 mm was significantly higher than in those with larger CBD stones. The differences of SO amplitudes and frequencies were not significant.

**Table 2:** Levels of alanine aminotransferase, aspartate transaminase, alkaline phosphatase, and *γ*-glutamyl transpeptidase in patients with normal or elevated basal sphincter of Oddi pressure.

	Basal SO pressure		*P* value
	<40 mmHg (*n* = 33)	>40 mmHg (*n* = 43)	
ALT (u/L)	157.86 ± 167.18	105.66 ± 112.23	0.108
AST (u/L)	88.25 ± 107.66	57.61 ± 81.38	0.162
AKP (u/L)	250.64 ± 235.37	180.45 ± 132.37	0.104
*γ*-GT (u/L)	410.70 ± 426.02	305.13 ± 360.71	0.246

SO: sphincter of Oddi, ALT: alanine aminotransferase, AST: aspartate transaminase, AKP: alkaline phosphatase, and *γ*-GT: *γ*-glutamyl transpeptidase.

No significant levels of ALT, AST, *γ*-GT, and AKP in patients with normal or elevated basal SO pressure.

**Table 3:** Basal SO pressure, amplitude, and frequency in healthy volunteers.

Enrolled numbers	Basal SO pressure (mmHg)	SO amplitude (mmHg)	SO frequency (/min)	Reference
43	13.4 ± 6.2	57.2 ± 6.7	Not listed	Gregg and Carr-Locke [13]
25	Not listed	57.2 ± 10.7	5.6 ± 2.4	Carr-Locke and Gregg [14]
